# A cyst in the mist: bronchogenic mediastinal cysts

**DOI:** 10.1093/jscr/rjad020

**Published:** 2023-01-31

**Authors:** Sowmya Prasanna Kumar Menon, Lianne Wachira, Lakshmi Srinivasan

**Affiliations:** School of Medicine, Keele University, Keele, Staffordshire, UK; Cardiothoracic Department, University Hospital North Midlands, Stoke-On-Trent, Staffordshire, UK; Cardiothoracic Department, University Hospital North Midlands, Stoke-On-Trent, Staffordshire, UK

## Abstract

Malformations of the bronchopulmonary foregut can lead to the formation of bronchogenic mediastinal cysts (BMC). BMC are rare congenital malformations usually found in the middle or posterior mediastinum. Only one-third of patients with BMC are symptomatic. We report a case of BMC in a 48-year-old female who was referred to the cardiothoracic surgeons due to an incidental finding of an anterior mediastinal mass on investigation for intermittent chest pain. The mass was treated surgically with a partial median sternotomy and mass excision. The patient’s symptomology and mass histology were atypical for BMC. At follow-up, the patient reported no residual symptoms. This case demonstrates the significance of considering BMC, especially the anterior subtype, as a differential diagnosis in the findings of patients with intermittent chest pain and computerized tomography findings of a mediastinal mass.

## INTRODUCTION

Malformations of the bronchopulmonary foregut can occur in gestational weeks 4–6, which may lead to bronchogenic mediastinal cysts (BMC) [1, 2]. BMC are closed sacs, originating from cells that are isolated from the main pulmonary branching as the lung bud differentiates from the primitive gut. The location is dependent on the stage of the developmental pathway this occurs. The wall is lined by ciliated epithelium comprising hyaline cartilage, smooth muscle and bronchial glands [3]. These sacs are fluid-filled with protein, blood products and calcium oxalate, mimicking a solid lesion [4]. They are commonly found in the middle or posterior mediastinum in relation to the tracheobronchial tree. They can commonly be misdiagnosed as other types of mediastinal mass if it is discovered in an ectopic location [1, 5]. BMC has a prevalence of 1 per 42 000 and accounts for 50–60% of all mediastinal cysts [2]. Two-thirds of patients with BMC are asymptomatic [6]. We describe a rare case of an anterior BMC, in a middle-aged female presenting with symptoms uncharacteristic of BMC.

## CASE REPORT

A 48-year-old female was referred to the cardiothoracic unit for a suspicious anterior mediastinal mass. Nine months prior, she presented to the emergency department with left-sided facial, upper and lower limb paraesthesia, pain, weakness and 2-week history of sore throat. She denied dysphasia or dysphagia. Neurological examination was normal and throat examination revealed tonsillar exudates. Non-contrast computerized tomography (CT) head noted no active disease. The patient was commenced on Penicillin V for tonsillitis and aspirin for suspected transient ischaemic attack (TIA). She was referred to the TIA clinic for further investigation where her National Institute of Health Stroke Scale score and Rankin score was 0, making vascular aetiology unlikely.

**Figure 1:**
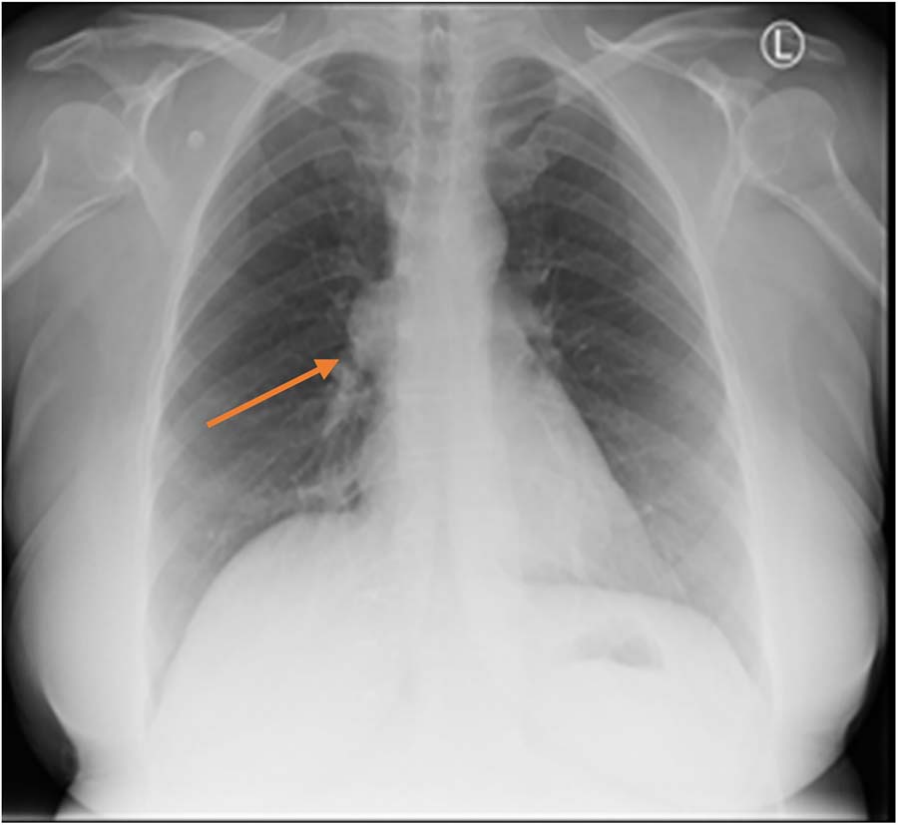
Chest X-ray showing anterior BMC (arrow).

**Figure 2:**
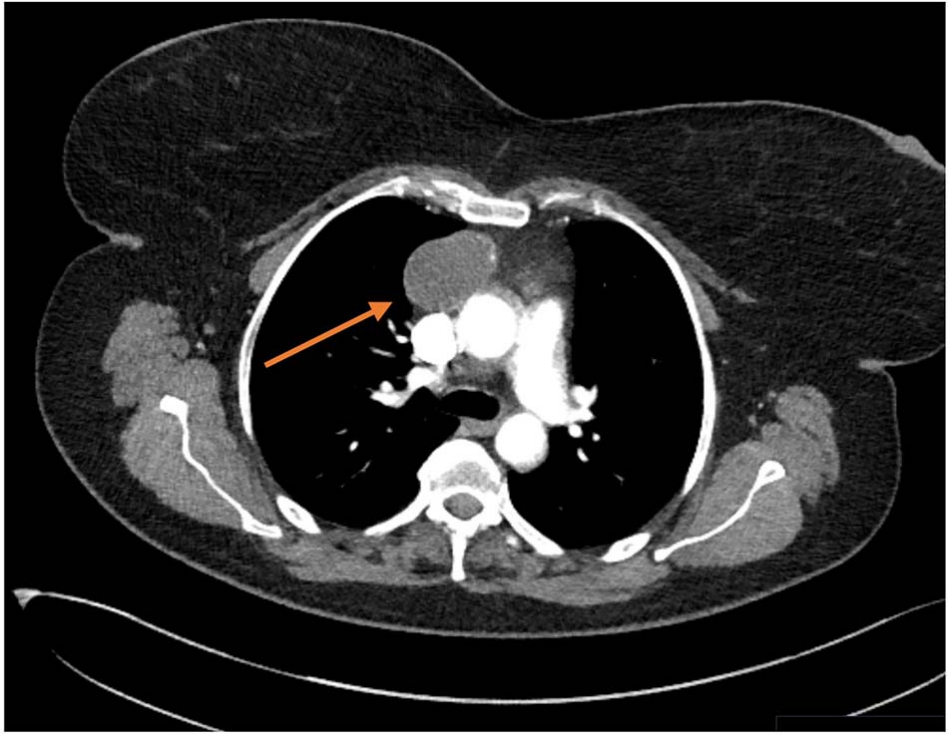
Transverse CT thorax showing anterior BMC (arrow).

Three months later the patient re-presented with intermittent chest pain associated with a 2-day history of non-productive cough. She was investigated with a chest X-ray and then a contrast CT chest (Figs 1 and 2). Contrast CT chest demonstrated a 20 mm × 40 mm large, slightly lobulated, oval-shaped right anterior mediastinal mass placed more in the superior mediastinum (Fig. 2). The appearance suggested a cystic density within the patchy calcification in the capsule wall. The multidisciplinary team concluded the mass was a thymic or pericardial cyst and planned for surgical excision if there was any progression of the mass.

A repeat contrast CT 5 months later confirmed no progression. However, the patient reported increasing chest pain and breathlessness and was referred for elective excision. The patient underwent partial median sternotomy to the fourth intercostal space, thymectomy and mediastinal mass excision. The aorta, brachiocephalic trunk, superior vena cava and thymus along with the superior and inferior cornu were dissected. The hard and nodular mass was situated on the right side of the anterior mediastinum and was adherent to the right upper lobe and phrenic nerve.

Histopathology of the mass showed a unilocular cyst lined by ciliated and non-ciliated columnar to cuboidal epithelium with focal squamous metaplasia. The cyst wall showed focal hyaline fibrosis and calcification and contained smooth muscle, bronchial mucous glands and cartilage. There was patchy chronic inflammation with granular eosinophilic material and scattered cholesterol clefts on the cyst wall in keeping with BMC. The surrounding fatty tissue contained small lobules of benign thymic tissue showing minimal focal nodular epithelial hyperplasia, essentially a normal thymus. There was no evidence of granulomatous inflammation of malignancy.

Post-operative recovery was uneventful, with no signs of infection. During the 2-week follow-up, the patient denied any shortness of breath or any restriction on activities of daily living.

## DISCUSSION

Bronchogenic cysts are rare congenital defects present due to the abnormal germination of the embryonic foregut that results in a fluid-filled pouch instead of normal bronchial development [5]. Most commonly, they arise due to abnormal budding along the tracheobronchial tree during embryonic development [1]. In adults, bronchogenic cysts are an incidental radiological finding with two-thirds of patients being asymptomatic [1, 2]. It accounts for 50–60% of mediastinal cysts and up to 15% of mediastinal tumours [2]. 79% of BMC are found in the middle mediastinum, 17% in the posterior mediastinum and 3% in the anterior mediastinum. Within the middle mediastinum, paratracheal and tracheal carina are the most common locations [7]. The commonest cystic lesions in the anterior mediastinum are pericardial (9%) and thymic (13%) in origin [4]. Although rare, BMC can be symptomatic due to cyst infection or compression of nearby structures, leading to respiratory distress and cough [8]. These cysts have a small chance of becoming malignant if left untreated [9]. Contrast CT chest is the investigation of choice for suspected BMC, presenting as homogenous, smooth round masses on the middle mediastinum with rare incidence of calcification [5, 8]. Interestingly in this case, the contrast CT thorax demonstrated a large, slightly lobulated, oval-shaped right anterior mediastinal mass with the appearance suggesting a cystic density with patchy calcification in the capsule wall. Histopathological examination can provide a definitive diagnosis and exclude the other differentials. The cyst wall had focal hyaline fibrosis and calcification and contained smooth muscle, bronchial mucous glands and cartilage and lined by ciliated and non-ciliated columnar to cuboidal epithelium, in keeping with BMC. Our patient represents a rare case of BMC due to the presentation and location [8]. Cases of BMC have been reported in literature with majority looking at the mediastinal subtype [10].

The management of those with bronchogenic cyst relies on the symptoms of the patient. Thoracotomy or video-assisted thoracoscopic surgery can be used to resect cysts that are symptomatic. Management of asymptomatic cysts is debated, but surgical management is preferred [11]. Thoracotomy is preferred for patients with complicated bronchogenic cysts [4]. In our case, due to the lobulated nature of the cyst a sternotomy was performed, and the outcome resulted in symptom relief for the patient. Therefore, this case highlights that symptomatic anterior mediastinal bronchogenic cysts can occur but are uncommon. The occurrence of ectopic bronchogenic cysts in the anterior mediastinum are documented in literature and should be considered in differential diagnosis.

## CONFLICT OF INTEREST STATEMENT

None declared.

## FUNDING

None.
